# The independent effects of vitamin D deficiency and house dust mite exposure on lung function are sex-specific

**DOI:** 10.1038/s41598-017-15517-z

**Published:** 2017-11-09

**Authors:** Nailê K. Nuñez, Ellen Bennett, Ling Chen, Paulo Márcio Pitrez, Graeme R. Zosky

**Affiliations:** 1 School of Medicine, Faculty of Health, University of Tasmania, Hobart, Tasmania Australia; 2 Laboratory of Pediatric Respirology, Infant Center, Institute of Biomedical Research, Pontifícia Universidade Católica do Rio Grande do Sul, Porto Alegre, Rio Grande do Sul Brazil

## Abstract

Vitamin D deficiency is increasing around the world and has been associated with the development of asthma. This study aims to evaluate the effect of dietary vitamin D deficiency at different life stages on lung function using a murine model of allergic airways disease. BALB/c mice were challenged intranasally with HDM or saline alone for 10 days. Twenty four hours after the last challenge, mice were anesthetized and lung function was measured using the forced oscillation technique (FOT). Mice were euthanized for assessment of inflammation in the bronchoalveolar lavage (BAL) and total collagen content in lung homogenates by ELISA. Vitamin D deficiency impaired lung function in both male and female mice, increasing tissue damping and elastance, however had no effect on HDM induced inflammation. The impact of vitamin D deficiency was more evident in females. HDM also decreased airway distensibility, but only in females and this response was not altered by vitamin D deficiency. Our data suggest that vitamin D deficiency and HDM exposure have independent effects on lung mechanics and that females are more susceptible to these effects. Vitamin D deficiency may exacerbate lung function deficits by having a direct, but independent, effect on parenchymal mechanics.

## Introduction

Asthma is a chronic disease characterized by airway inflammation, airway remodeling and reversible deficits in lung function^1,2^. The prevalence of asthma increases when communities adopt western lifestyles and become more urbanized^3–6^. Due to the associated reduction in outdoor activity, some have suggested that vitamin D deficiency may be responsible for this association^5^. It has been estimated that one billion people around the world have inadequate levels of vitamin D due to many factors such as an indoor life style, increased use of sunscreen^7^ and low dietary vitamin D^8^. Due to the scale of this problem, it is important that we understand the potential health implications of widespread vitamin D deficiency.

While recent vitamin D supplementation trials in community based cohorts of pregnant women have shown no effect on the risk of wheeze in children at 3 years of age^9,10^, it is unclear whether maternal vitamin D supplementation has effects on postnatal lung function, which is an important risk factor for asthma later in life^11^. We have shown that maternal vitamin D deficiency at 16–20 weeks’ gestation is associated with impaired lung function at 6 years of age in offspring^12^. In line with this finding, we have also shown that *in utero* vitamin D deficiency is sufficient to induce increased airway smooth muscle (ASM) mass and cause deficits in lung function in a mouse model^13–15^; both of which are key characteristics of the asthmatic phenotype^1^.

While these observations point to a role for vitamin D deficiency in causing alterations in lung structure, the inflammatory process itself can also lead to airway remodeling. House dust mite (HDM), a prevalent environmental allergen, is associated with allergic airway diseases^16^ and drives inflammatory processes that are associated with airway remodeling^17,18^ resulting in increased airway resistance^19^. HDM induces a robust Th-2 driven inflammatory response in the airways that is characterized by eosinophilia and the production of IL-4, IL-5 and IL-13^17,18^. These inflammatory processes lead to goblet cell metaplasia, an increase in ASM thickness and deposition of collagen around the airways^18,20^. Collectively, these structural changes cause deficits in lung function^18,19,21^.

Given that both vitamin D deficiency and HDM may lead to deficits in lung function, we investigated the interaction between vitamin D deficiency and HDM, and their effects on lung function. We hypothesized that the combination of vitamin D deficiency and HDM exposure would lead to deficits in lung function that are greater than the individual effects of vitamin D deficiency and HDM alone. We addressed this hypothesis by evaluating the effects of *in utero*, postnatal and whole life vitamin D deficiency on lung function in a murine model of HDM induced allergic airways disease.

## Materials and Methods

### Mouse model

All studies were conducted with the approval of the University of Tasmania Animal Ethics Committee and conformed to the guidelines of the National Health and Medical Research Council (Australia). Three-week old female BALB/c mice (Cambridge Farm Facility, University of Tasmania, TAS, AU) were placed on vitamin D deficient or replete diets and mated with vitamin D replete males at 8 weeks of age as described previously^14^. Pups were cross fostered at birth to assess the effects of *in utero* (Vit D −/+), postnatal (Vit D +/−) and whole-life (Vit D −/−) vitamin D-deficiency on inflammation and lung function outcomes compared to replete controls (Vit D +/+)^15^. At 8 weeks of age (7–13 mice per group; see Figure legends for further details), male and female offspring were challenged intranasally with 25 µg of an HDM extract (Greer Laboratories, Lenoir, NC, USA) in 50 µl of saline or saline alone for 10 consecutive days under light methoxyflurane anesthesia. Twenty four hours after the last challenge, the outcomes described below were assessed.

### Lung function

Mice were anesthetized with ketamine (40 mg/mL) and xylazine (2 mg/mL) by intraperitoneal injection at a dose of 0.01 mL/g body weight. Two-thirds of the dose was administered before tracheostomy and cannulation, and the remaining anesthetic was given when the mice were connected to the animal ventilator (HSE-Harvard MiniVent; Harvard Apparatus, Holliston, MA, USA). Mice were ventilated at 400 breaths/min with a tidal volume of 10 mL/kg, and 2 cmH_2_O of positive end-expiratory pressure (PEEP). Lung mechanics were assessed using a modified low frequency forced oscillation technique (LFOT)^22^ during slow inflation manoeuvers from end-expiratory lung volume (EELV), up to 20 cmH_2_O transrespiratory pressure (Prs)^22^. The oscillatory signal consisted of 9 frequencies ranging from 4–38 Hz and was delivered to the endotracheal cannula via a wavetube of known impedance to calculate the respiratory system impedance. A four-parameter model with constant-phase tissue impedance was fitted to the respiratory system impedance spectrum^23^. This allowed us to calculate airway resistance (R_aw_), tissue damping (G), tissue elastance (H) and hysteresivity (η = G/H) from 0 to 20 cmH_2_O Prs. We also used these data to calculate airway distensibility as the slope of the conductance (G_aw_ = 1/R_aw_) versus pressure curve between 2 and 10 cmH_2_O Prs.

### Differential cell counts

After lung function measurements, mice were euthanized with an overdose of ketamine/xylazine, and a bronchoalveolar lavage (BAL) was performed by washing the lung 3 times with 500 µL of saline. The BAL was centrifuged at 5000 rpm for 5 minutes. Cytospin slides were generated from the resuspended pellet and stained with Haem Kwik (HD Scientific Supplies Pty Ltd., AU). Differential cells counts were performed under light microscopy by counting a minimum of 200 cells per mouse.

### Collagen

We have previously found that vitamin D deficiency can increase collagen type 1 alpha 1 (COL1A1) expression *in utero* ^24^ which may impact on lung mechanics. In order to determine whether this persisted into adulthood, and whether it was altered by HDM exposure, we assessed COL1A1 expression in lung homogenates by ELISA according to the manufacturer’s instructions (DLDEVELOP Ltd., Wuxi, Jiangsu, PRC). COL1A1 levels were calculated relative to total protein content in the lung measured by Bradford assay (Thermo Fisher Scientific, Whaltham, MA, USA).

### Statistical analysis

SigmaPlot (v12.5, Systat, Germany) was used to perform the statistical analysis. Two-way ANOVA with Holm-Sidak posthoc tests were used to assess the effect of vitamin D and HDM exposure on the outcomes of interest. Data were log-transformed when necessary to satisfy the model assumptions. A p-value < 0.05 was considered significant. Data are presented as mean (SD).

## Results

### Lung function

**Figure 1:**
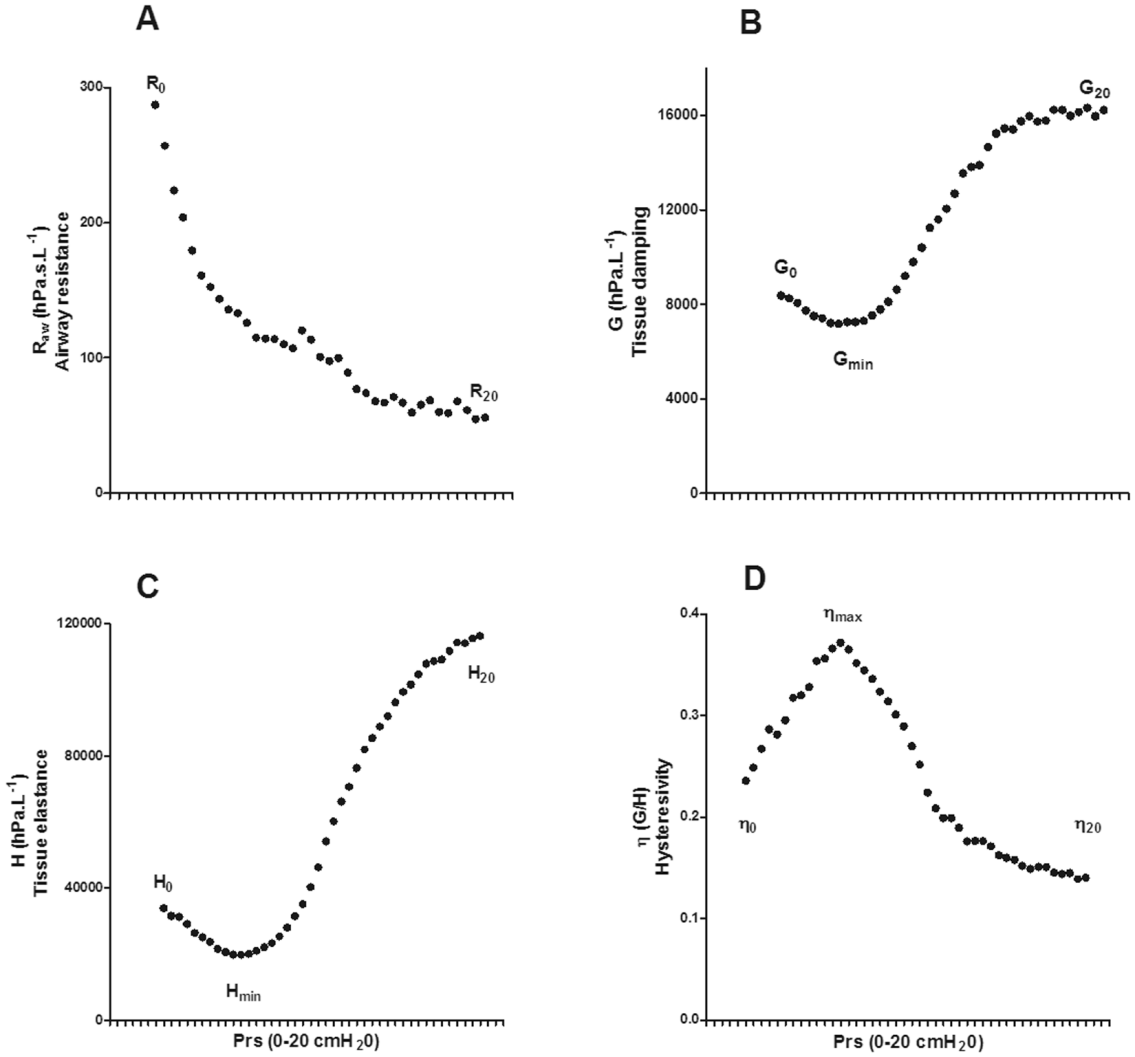
Mean R_aw_ (**A**; Newtonian resistance ~ airway resistance), G (**B**; tissue damping), H (**C**; tissue elastance) and η (**D**; hysteresivity) plotted against transrespiratory pressure (Prs) for female vitamin D replete (Vit D +/+) mice showing the characteristic pressure dependence of these parameters. In order to simplify the subsequent comparisons between groups we represented these curves by the indices indicated in the graphs; R_0_, R_20_, G_0_, G_min_, G_20_, H_0_, H_min_, H_20_, η_0_, η_max_, η_20_.

R_aw_, G, H and η have characteristic pressure dependences^25^. Specifically, R_aw_ (airway resistance) decreases monotonically from 0 to 20 cmH_2_O Prs (Fig. 1A), G (tissue damping) and H (tissue elastance) (Fig. 1B,C) initially decrease as Prs increases before increasing exponentially at high Prs, while η (hysteresivity = G/H; Fig. 1D) initially increases before decreasing at high Prs. In order to simplify the analysis of our data, and to facilitate simple comparisons between groups, we characterized the pressure dependence of lung mechanics using the following indices: R_aw_, G, H and η at 0 cmH_2_O Prs (R_0_, G_0_, H_0_, η_0_), R_aw_, G, H and η at 20 cmH_2_O Prs (R_20_, G_20_, H_20_, η_20_), the minimum G and H (G_min_, H_min_) and the maximum η (η_max_) (Fig. 1). We then compared these parameters for each of the vitamin D deficiency groups (Vit D −/−, Vit D −/+ and Vit D +/−) against the replete controls (Vit D +/+).

#### Females

**Figure 2:**
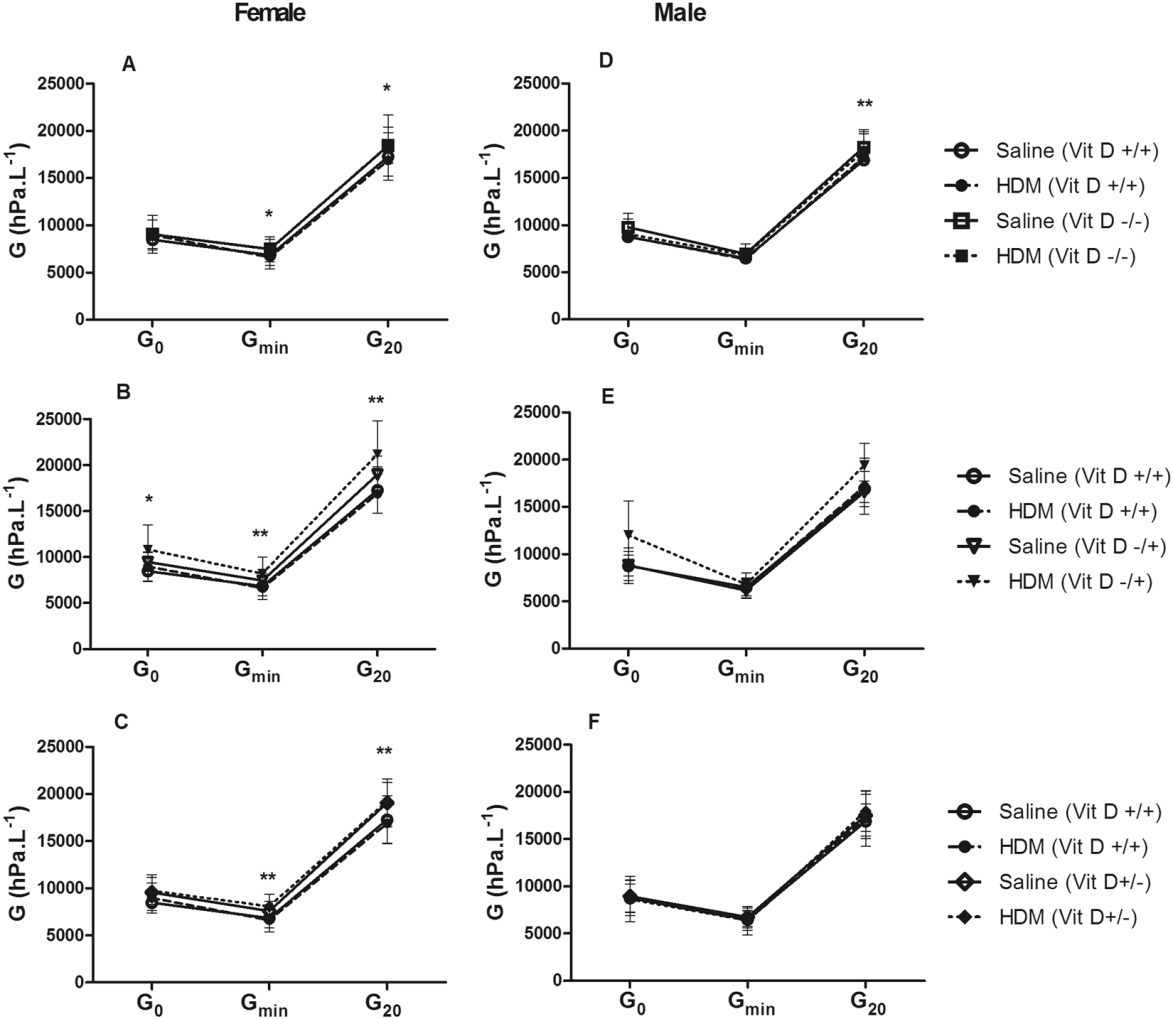
Tissue damping (G) at 0 cmH_2_O Prs (G_0_), 20 cmH2O Prs (G_20_) and the minimum (G_min_) for female (**A**–**C**) and male (**D**–**F**) saline and house dust mite (HDM) exposed mice that were vitamin D replete (Vit D +/+), whole-life vitamin D deficient (Vit D −/−; **A**,**D**), *in utero* vitamin D deficient (Vit D −/+; **B**,**E**) or post-natal vitamin D deficient (Vit D +/−; **C**,**F**). Data are presented as mean (SD), n = 9–13 for each group. *p < 0.05; **p < 0.01; ***p < 0.001.

**Figure 3:**
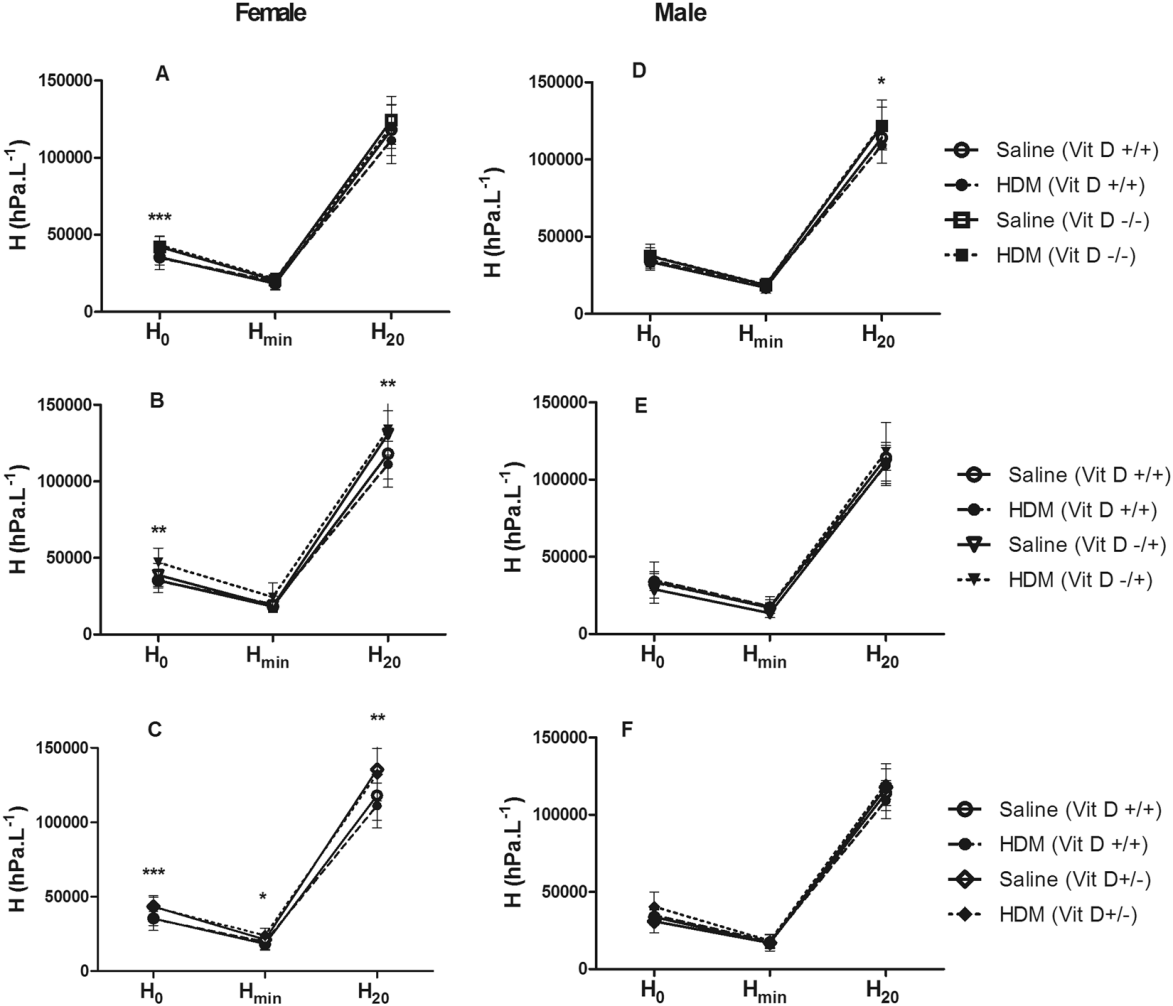
Tissue elastance (H) at 0 cmH_2_O Prs (H_0_), 20 cmH2O Prs (H_20_) and the minimum (H_min_) for female (**A**–**C**) and male (**D**–**F**) saline and house dust mite (HDM) exposed mice that were vitamin D replete (Vit D +/+), whole-life vitamin D deficient (Vit D −/−; **A**,**D**), *in utero* vitamin D deficient (Vit D −/+; **B**,**E**) or post-natal vitamin D deficient (Vit D +/−; **C**,**F**). Data are presented as mean (SD), n = 9–13 for each group. *p < 0.05; **p < 0.01; ***p < 0.001.

**Figure 4:**
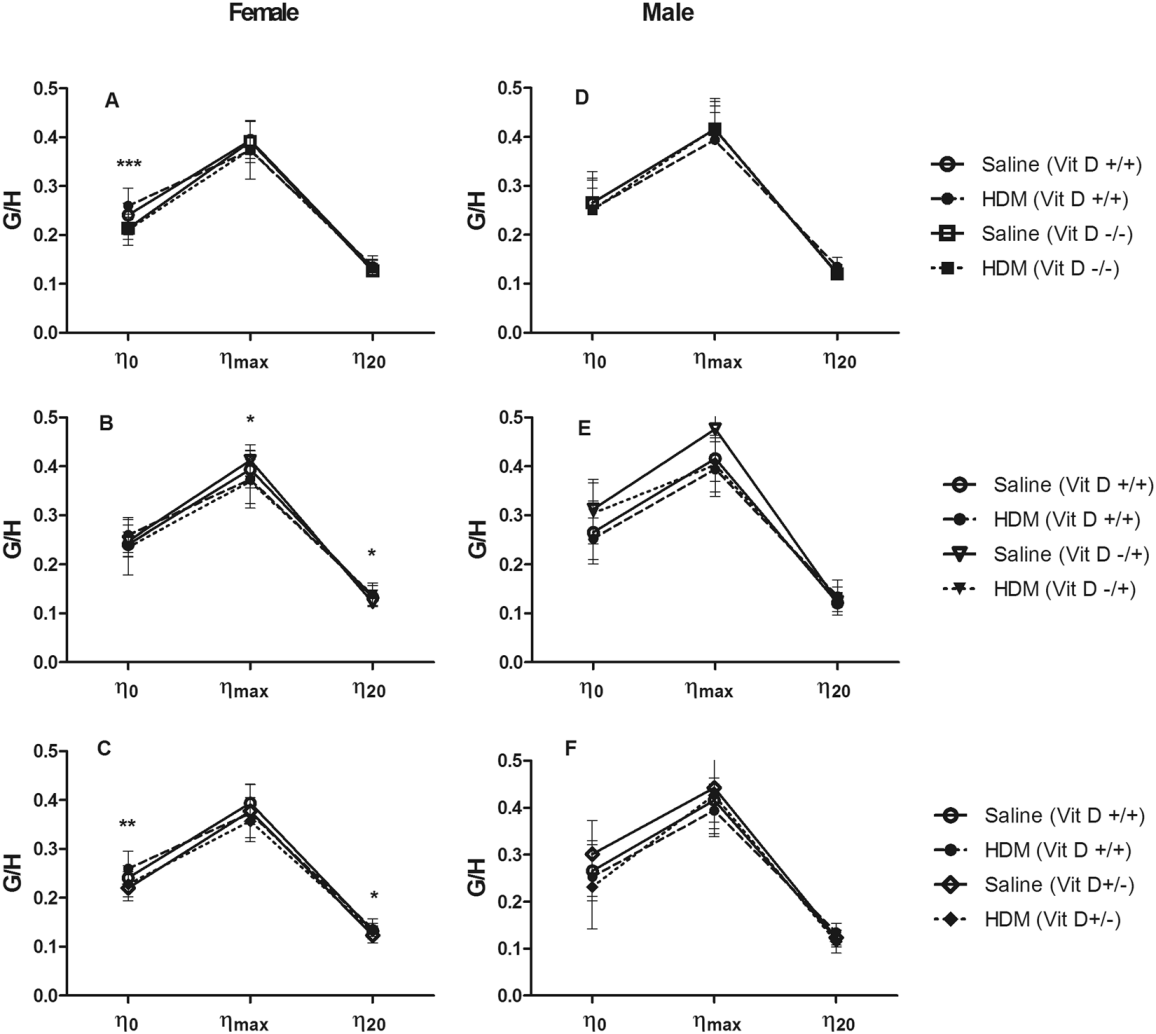
Hysteresivity at 0 cmH_2_O Prs (η_0_), 20 cmH2O Prs (η_20_) and the maximum (η_max_) for female (**A**–**C**) and male (**D**–**F**) saline and house dust mite (HDM) exposed mice that were vitamin D replete (Vit D +/+), whole-life vitamin D deficient (Vit D −/−; **A**,**D**), *in utero* vitamin D deficient (Vit D −/+; **B**,**E**) or post-natal vitamin D deficient (Vit D +/−; **C**,**F**). Data are presented as mean (SD) n = 9–13 for each group. *p < 0.05; **p < 0.01; ***p < 0.001.

**Figure 5:**
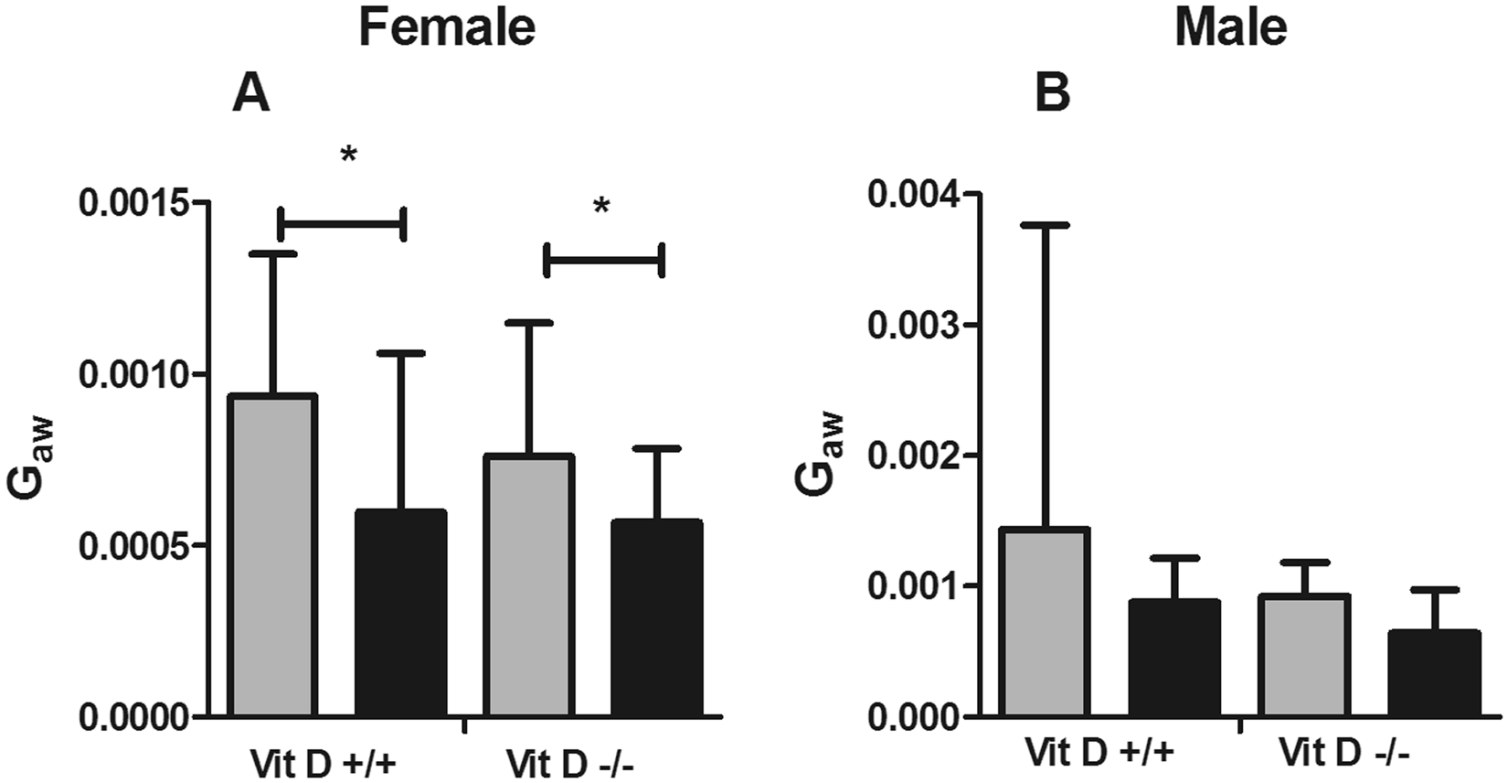
Airway distensibility, calculated as the slope of the conductance (G_aw_ = 1/R_aw_) versus pressure curve between 2 and 10 cmH_2_O Prs, for female (**A**) and male (**B**) vitamin D replete (Vit D +/+) and vitamin D deficient (Vit D −/−) mice exposed to 25 µg of HDM in 50 µL intranasally for 10 days (black bars) or saline alone (grey bars). Data are presented as mean (SD), n = 7–10 for each group in female and 8–12 in male. *p < 0.05.

In females, R_aw_ was not affected by HDM (p > 0.05 for all comparisons) or vitamin D deficiency (p > 0.05 for all comparisons, *data not shown*). However, whole-life vitamin D deficiency increased tissue damping (Fig. 2A) at G_min_ (7470 hPa.L^−1^ vs 6730 hPa.L^−1^; p = 0.027) and G_20_ (18460 hPa.L^−1^ vs 17090 hPa.L^−1^; p = 0.046), tissue elastance (Fig. 3A) at H_0_ (42730 hPa.L^−1^ vs 35210 hPa.L^−1^; p < 0.001) and decreased hysteresivity (Fig. 4A) at η_0_ (0.21 vs 0.25; p < 0.001). Many of these deficits in lung mechanics were also evident in female mice that were only vitamin D deficient *in utero* or postnatally. *In utero* vitamin D deficiency increased tissue damping (Fig. 2B) at G_0_ (10150 hPa.L^−1^ vs 8720 hPa.L^−1^; p = 0.023), G_min_ (7820 hPa.L^−1^ vs 6730 hPa.L^−1^; p = 0.009) and G_20_ (20080 hPa.L^−1^ vs 17090 hPa.L^−1^; p = 0.003), tissue elastance (Fig. 3B) at H_0_ (42990 hPa.L^−1^ vs 35210 hPa.L^−1^; p = 0.005) and H_20_ (132500 hPa.L^−1^ vs 114640 hPa.L^−1^; p = 0.002) and decreased the hysteresivity (Fig. 4B) at η_max_ (0.39 vs 0.38; p = 0.037) and η_20_ (0.39 vs 0.38; p = 0.025). Postnatal vitamin D deficiency increased tissue damping (Fig. 2C) at G_min_ (7830 hPa.L^−1^ vs 6730 hPa.L^−1^; p = 0.007) and at G_20_ (19160 hPa.L^−1^ vs 17090 hPa.L^−1^; p = 0.009), tissue elastance (Fig. 3C) at H_0_ (43260 hPa.L^−1^ vs 35210 hPa.L^−1^; p < 0.001), H_min_ (22540 hPa.L^−1^ vs 19000 hPa.L^−1^; p = 0.023) and at H_20_ (133770 hPa.L^−1^ vs 114640 hPa.L^−1^; p = 0.004) and decreased the hysteresivity (Fig. 4C) at η_0_ (0.22 vs 0.25; p = 0.006) and at η_20_ (0.13 vs 0.14; p = 0.042). House dust mite had no effect on these measures of lung mechanics (p > 0.05 for all comparisons). In contrast, HDM decreased airway distensibility (p = 0.036, Fig. 5), a measure of airway stiffness, in female whole-life vitamin D deficient, while vitamin D deficiency had no effect on airway distensibility (p > 0.05).

#### Males

In males, whole-life vitamin D deficiency increased airway resistance at R_0_ (p = 0.017, *data not shown*), tissue damping at G_20_ (18070 hPa.L^−1^ vs 17040 hPa.L^−1^; p = 0.008) (Fig. 2D) and, tissue elastance at H_20_ (122340 hPa.L^−1^ vs 111740 hPa.L^−1^; p = 0.011) (Fig. 3D), with no differences in hysteresivity (Fig. 4D). Similar to the female mice, HDM exposure did not affect R_aw_, G, H or η (p > 0.05 for all comparisons). In contrast to the female mice, deficits in lung mechanics were only observed in male mice that were whole-life vitamin D deficient, while airway distensibility (Fig. 5B) was not affected by HDM exposure (p = 0.48) or vitamin D deficiency (p = 0.85).

### Differential cell counts

#### Females

**Figure 6:**
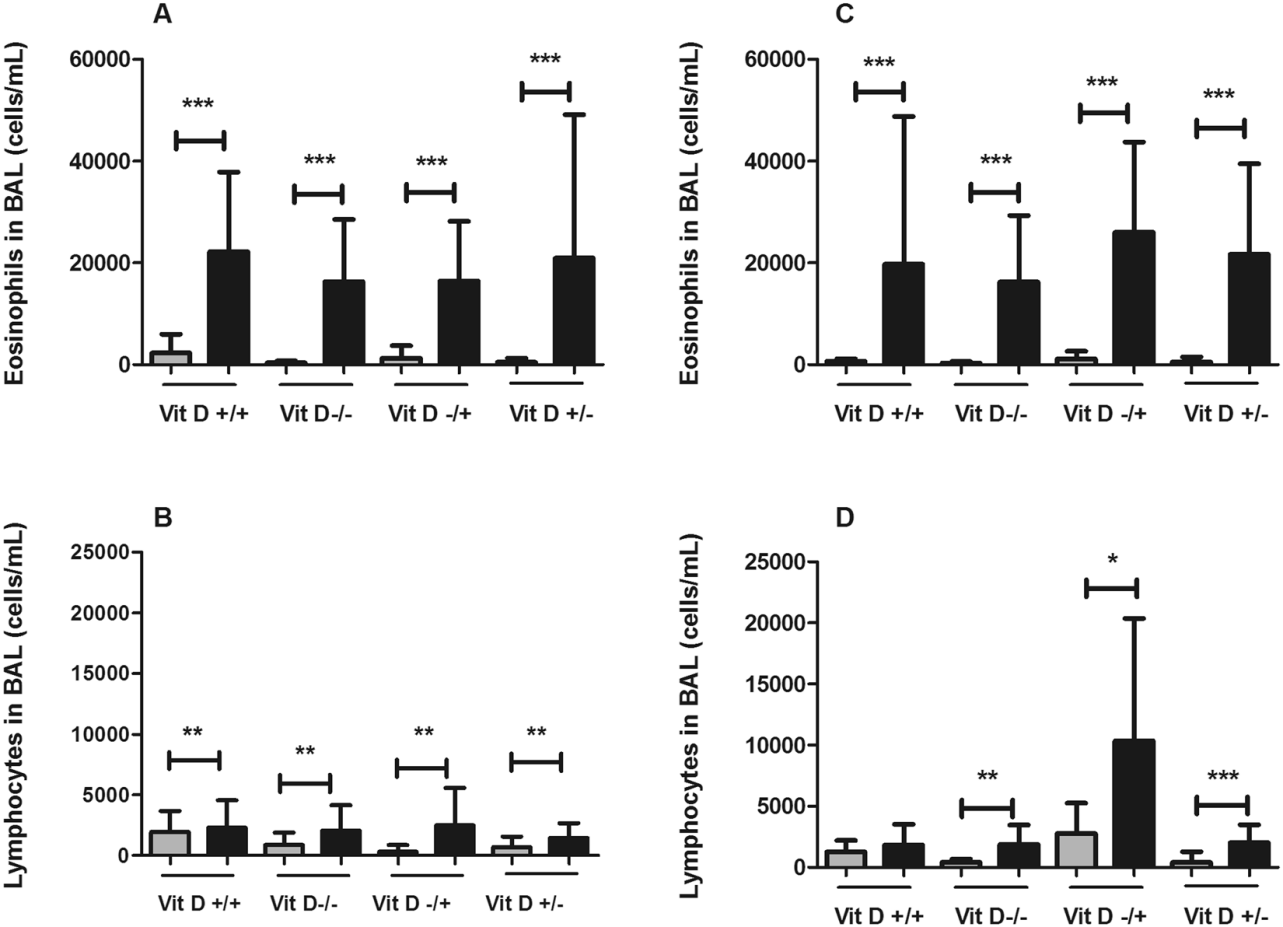
Eosinophil (**A**,**C**) and lymphocyte (**B**,**D**) cell counts in the BAL of female (**A**,**B**) and male (**C**,**D**) mice that were whole-life replete (Vit D +/+), whole-life deficient (Vit D −/−), *in utero* deficient (Vit D −/+) or postnatally deficient (Vit D +/−) in vitamin D and exposed to 25 µg of house dust mite (HDM; black bars) intranasally in 50 µL of saline or saline alone (grey bars) for 10 consecutive days. Data are presented as mean (SD), n = 8–11 for each group in females and 7–11 in males. *p < 0.05; **p < 0.01; ***p < 0.001.

In females, HDM caused an influx of eosinophils (p < 0.001) and lymphocytes (p = 0.008) in the BAL (Fig. 6A,B), however vitamin D deficiency had no effect on the HDM induced influx of eosinophils (p = 0.811) or lymphocytes (p = 0.320). Neutrophil and macrophage numbers were not altered by vitamin D deficiency (neutrophils, p = 0.928; macrophages, p = 0.157) or by HDM (neutrophils, p = 0.631; macrophages, p = 0.231) (*data not shown*).

#### Males

In males, HDM caused an influx of eosinophils (p < 0.001, Fig. 6C) and neutrophils (p < 0.001, *data not shown*), while vitamin D deficiency had no effect on these cells (eosinophils, p = 0.761; neutrophils, p = 0.550). In contrast to the female mice, HDM also increased lymphocytes numbers in the BAL (Fig. 6D), but only in the groups that were vitamin D deficient (Vit D −/− p = 0.003, Vit D −/+ p = 0.012 and Vit D +/− p < 0.001). Macrophage numbers in the BAL were not affected by vitamin D (p = 0.125) or HDM (p = 0.779) in male mice (*data not shown)*.

### Collagen

We sought to determine whether the effects we saw were due to differences in COL1A1 expression, however there were no differences in COL1A1 between groups (*data not shown*).

## Discussion

In this study, we evaluated the independent and combined effect(s) of vitamin D deficiency during different life stages (*in utero* and/or postnatal) and allergen exposure on lung function outcomes using a mouse model. Vitamin D deficiency *in utero* and/or postnatally had wide-ranging effects on lung function, particularly in female mice, causing significant impairments in tissue mechanics (G, H and G/H). Vitamin D deficiency also resulted in an impairment in lung mechanics (R_aw_, G, and H) in male mice, but to a lesser extent than observed in females, and only in response to whole-life vitamin D deficiency. Vitamin D deficiency did not appear to have an influence on airway stiffness. In contrast, while HDM had no effect on the pressure dependence of R_aw_, G or H it significantly decreased airway stiffness; but only in female mice. These differences were discordant with cellular inflammation and could not be explained by differences in collagen expression in the lungs. These findings suggest that vitamin D deficiency and HDM have independent effects on lung function, which are unrelated to inflammation, and are sex-dependent. Thus, the net effect of *in utero* vitamin D deficiency on lung outcomes may depend on whether you are female or male, and will be influenced by the effect of postnatal allergen responses via vitamin D independent pathways.

In this study, vitamin D deficiency had a significant impact on lung mechanics, particularly parenchymal tissue mechanics. In males these effects were limited to the whole-life vitamin D deficiency group while in females these deficits were evident in all deficient groups. The observation that whole-life vitamin D deficiency increased G, a measure of lung mechanics linked to the small airways and ventilation heterogeneity^26^, in both males (at G_20_) and females (G_min_ and G_20_) at 8 weeks of age is consistent with our previous studies on 2 weeks old animals^13^. A similar pattern was observed in H, a measure of tissue stiffness, while changes in η were only observed in females. Collectively, these observations suggest that vitamin D deficiency has an impact on parenchymal lung mechanics. Given that we have previously shown that vitamin D deficiency does not affect lung volume in adulthood^14^, it is unlikely that these differences are due to the influence of vitamin D deficiency on somatic growth. Vitamin D deficiency in female mice, either *in utero* or postnatally, was sufficient to impair lung function. These deficits in lung function may increase susceptibility to chronic lung disease and respiratory morbidity later in life^27^.

There are well described differences in lung mechanics between males and females and we have previously described increased susceptibility in females to altered lung function as a result of *in utero* vitamin D deficiency^12,15^. In relation to asthma, boys have a higher prevalence of asthma in early life whereas, after puberty, asthma is more prevalent in females^28^. Similarly, airway reactivity increases with age in females but decreases in males^29^. Some of these sex differences in asthma susceptibility have been linked to estrogen levels^30^ and estrogen signaling is a critical component of lung development^31^. Given the intimate association between vitamin D and estrogen synthesis^32^, it is possible that estrogen is related to the increased susceptibility of females to the effects of vitamin D deficiency; although we did not directly address this in the present study.

Despite the strong impact of vitamin D deficiency on the pressure-dependent parenchymal mechanics, airway distensibility was not affected by vitamin D deficiency. However, airway distensibility was diminished after exposure to HDM, but only in whole-life vitamin D deficient female mice. Airway distensibility is related to airway stiffness and is reduced in asthmatics^33^. Our observation provides evidence that HDM exposure can directly alter airway stiffness, which has been linked to the increased propensity of the asthmatic airway to constrict^34^. Based on these data, it is clear that vitamin D and HDM had independent effects on lung function whereby vitamin D did not modify the response to HDM for any of the lung function outcomes we measured. Thus, the net effect of HDM exposure and vitamin D deficiency is likely to be additive.

Interestingly, these lung function responses were completely discordant with inflammation. For example, in female mice, HDM caused substantial eosinophilia that was unaltered by vitamin D deficiency and yet parenchymal mechanics was altered in response to vitamin D deficiency. In contrast, vitamin D deficiency modified the inflammatory response to HDM in male mice but this was not associated with alterations in lung mechanics. While eosinophilia was associated with decreased airway distensibility in the females, inflammation was clearly not sufficient to alter airway stiffness in all cases as this association was not evident in male mice. At this stage we also do not have a structural explanation for the alterations in lung function and deficits in parenchymal mechanics as a result of vitamin D deficiency were not due to altered type 1 collagen levels.

There are several limitations to this study. Firstly, after the measurements of lung function and post-mortem tissue processing (collecting BAL fluid), we were unable to obtain reliable structural measurements that could be directly related to the changes in lung function. Secondly, structural protein analysis was limited to only one type of collagen, but it is possible the changes in lung mechanics may be related to other functional proteins such as surfactant proteins that are essential for lung function and pulmonary homeostasis.

Notwithstanding these limitations, our data suggest that vitamin D deficiency and HDM have independent effects on lung function that are sex-specific. HDM induces a robust inflammatory response that may lead to increased airway stiffness in females. In contrast, vitamin D deficiency had limited effects on inflammation but caused consistent deficits in parenchymal mechanics that were more pronounced in female mice. Interestingly, *in utero* or postnatal vitamin D deficiency was sufficient to alter lung mechanics in these mice. While we were unable to identify mechanisms linking these observations, our data clearly highlight the complexity of the effects of vitamin D on lung function and the importance of probing the influence of sex on responses to respiratory insults.

### Data availability

All data generated or analysed during this study are included in this published article.
